# Cell targeting and immunostimulatory properties of a novel Fcγ-receptor-independent agonistic anti-CD40 antibody in rhesus macaques

**DOI:** 10.1007/s00018-023-04828-2

**Published:** 2023-06-23

**Authors:** Xianglei Yan, Sebastian Ols, Rodrigo Arcoverde Cerveira, Klara Lenart, Fredrika Hellgren, Kewei Ye, Alberto Cagigi, Marcus Buggert, Falk Nimmerjahn, Jesper Falkesgaard Højen, Daniel Parera, Ulrich Pessara, Stephan Fischer, Karin Loré

**Affiliations:** 1 Division of Immunology and Allergy, Department of Medicine Solna, Karolinska Institutet and Karolinska University Hospital, Visionsgatan 4, BioClinicum J7:30, 171 64 Stockholm, Sweden; 2 Center of Molecular Medicine, Stockholm, Sweden; 3 Department of Medicine Huddinge, Center for Infectious Medicine, Karolinska Institutet, Stockholm, Sweden; 4 Division of Genetics, Department of Biology, University of Erlangen-Nürnberg, Erlangen, Germany; 5 Department of Infectious Diseases, Aarhus University Hospital, Aarhus, Denmark; 6 Department of Clinical Medicine, Aarhus University, Aarhus, Denmark; 7 Department of Medicine, University of Colorado Denver, Aurora, CO USA; 8 Icano MAB GmbH, Polling, Germany

**Keywords:** Adjuvant, Vaccine, Innate immunity, Immunotherapy, Non-human primate

## Abstract

Targeting CD40 by agonistic antibodies used as vaccine adjuvants or for cancer immunotherapy is a strategy to stimulate immune responses. The majority of studied agonistic anti-human CD40 antibodies require crosslinking of their Fc region to inhibitory FcγRIIb to induce immune stimulation although this has been associated with toxicity in previous studies. Here we introduce an agonistic anti-human CD40 monoclonal IgG1 antibody (MAB273) unique in its specificity to the CD40L binding site of CD40 but devoid of Fcγ-receptor binding. We demonstrate rapid binding of MAB273 to B cells and dendritic cells resulting in activation in vitro on human cells and in vivo in rhesus macaques. Dissemination of fluorescently labeled MAB273 after subcutaneous administration was found predominantly at the site of injection and specific draining lymph nodes. Phenotypic cell differentiation and upregulation of genes associated with immune activation were found in the targeted tissues. Antigen-specific T cell responses were enhanced by MAB273 when given in a prime-boost regimen and for boosting low preexisting responses. MAB273 may therefore be a promising immunostimulatory adjuvant that warrants future testing for therapeutic and prophylactic vaccination strategies.

**Supplementary Information:**

The online version contains supplementary material available at 10.1007/s00018-023-04828-2.

## Introduction

In the past decade, development of targeted therapies that block immune checkpoint inhibitors has revolutionized immunotherapy. However, blocking immune checkpoints alone such as programmed cell death protein 1 (PD-1) or cytotoxic T-lymphocyte-associated protein 4 (CTLA-4) is not sufficient as most patients do not induce long-term sustained and effective responses [1–3]. Additional strategies to specifically target immune cells in order to enhance antigen presentation and T cell responses are being explored. One such strategy is targeting the CD40/CD40L interaction [4]. CD40 is a member of the tumor necrosis factor receptor superfamily (TNFRSF) predominantly expressed on the cell-surface of antigen presenting cells (APCs) including B cells, dendritic cells (DCs) and monocytes and works as a costimulatory receptor [5]. CD40 binds to CD40 ligand (CD40L, CD154) on T cells during the antigen presentation process which leads to strong activation of both APCs and T cells [6]. Previous studies have shown that CD40 activation can replace T cell help required to drive CD8 T cell responses [7–9]. CD40 agonists have, therefore, been of interest to develop as candidates for adjuvants or cancer immunotherapy [10].

Among the CD40 agonists that have progressed to clinical development, the monoclonal antibody CP-870,893 (Selicrelumab) is the most studied. It has been tested for treatment of several solid tumors, such as melanoma [11–14]. While it has been shown that a single dose can induce antitumor activity in some but not all of the individuals, the main dose-limited adverse events were cytokine release syndrome (CRS) with grade 1–2, transient liver function test abnormalities and transient decrease in platelet counts [11–13, 15]. More information is needed of the mechanisms by which CD40 agonists can mediate beneficial immune stimulation without unwanted side effects in order to refine CD40 agonists further. A proposed strategy to improve safety is to modify the fragment crystallizable region (Fc region) of CD40 antibodies. Among the Fc-gamma receptors (FcγRs), binding to FcγRIIb has been shown to potently enhance the activity of CD40 agonists [16–18]. The new generation of CD40 agonists with an engineered Fc region to increase binding to FcγRIIb has. Therefore. been developed [18–22]. However, by introducing mutations in CP-870,893 to increase the affinity to FcγRIIb side effects were increased [20, 21]. These results suggest that in addition to the canonical CD40 signaling leading to antigen-specific T cell licensing, alternative pathways such as FcγRIIb crosslinking are related to toxicity where mutations increasing binding to FcγRIIb have been introduced [20, 21]. Manipulating or reducing FcγRIIb or even FcγR crosslinking in general is one strategy to develop safe but potent CD40 agonistic antibodies.

MAB273 is a novel humanized rabbit IgG1 with the LALA (L234A and L235A)-mutations in the Fc region which disables Fcγ-receptor-mediated crosslinking [23]. In this study, we tested the CD40 binding and immune activation capacity of MAB273 in human and rhesus macaque peripheral blood mononuclear cells (PBMCs) and confirmed that this was independent of the Fc region. The tolerability and immunostimulatory functions of MAB273 in vivo were further tested in rhesus macaques by analyzing biodistribution in different tissues and cells, innate immune activation and induction of antigen-specific T cells.

## Materials and methods

### Antibodies and the generation of fragments

MAB273 (humanized rabbit IgG1 with LALA mutated Fc region), CP-870,893 (Selicrelumab, human IgG2 agonistic anti-CD40 monoclonal antibody) as well as the human IgG1 isotype control antibody with LALA mutations in the Fc region (IgG1-LALA) were provided by Icano MAB GmbH. The isotype control antibody IgG1-LALA was for MAB273. The isotype control antibody for CP-870,893 was not included. F(ab’)2 and Fab fragments of MAB273 were generated using the Pierce^™^ F(ab’)2 Preparation Kit and Pierce^™^ Fab Preparation Kit (ThermoFisher, 44988 and 44985) according to manufacturer’s instructions. After pepsin or papain digestion, and a first round of purification, the products were further purified using CH1-XL columns (ThermoFisher, A37054) recognizing the CH1 domain of human IgG as well as Fc-XP columns (ThermoFisher, 494371201) containing anti-IgG-CH3 matrix. A SDS-PAGE (Bio-Rad, 4568123) and an endotoxin detection assay (GenScript, L00350) were performed according to manufacturer’s instructions to verify the purity, correct valency and low endotoxin content, respectively.

### Human samples

The human blood samples (buffy coats) were obtained from anonymous healthy blood donors at the Karolinska University Hospital. The blood samples could not be linked to donor identities. The sample size is indicated in each figure legend.

### Animals

This study was approved by the Stockholm Regional Ethical Board on Animal Experiments. Six male Indian-origin rhesus macaques (for the toxicity and safety study) as well as three male and three female Indian-origin rhesus macaques (for the immunogenicity and biodistribution study) were housed at Astrid Fagraeus Laboratory, Karolinska Institutet. All procedures were performed according to the guidelines of the Association for Assessment and Accreditation of Laboratory Animal Care.

### Immunizations and sampling

In the initial toxicity and safety study, the animals were divided into three groups. Two animals received intravenous (i.v.) administration of 1 mg/kg anti-CD40 monoclonal antibody (MAB273), two animals received 0.1 mg/kg MAB273 by i.v., and the last two animals received subcutaneous (s.c.) administration of 0.1 mg/kg MAB273. For i.v. administration, an intravenous infusion of the antibody into the saphenous vein was performed. A total volume of 25 ml was slowly distributed stepwise for 30 min. For s.c. administration, a single 0.5 ml subcutaneous injection of the antibody in the skin above the left quad muscle was performed. Blood draws were performed at pre-dose, 30 min, 4 h, 24 h, 48 h, 72 h, 1 week, 2 weeks, 3 weeks, and 4 weeks after MAB273 administration in heparin tubes.

In the immunogenicity study, animals were divided into two groups. In the therapeutic vaccination group, animals were immunized s.c. 7 weeks apart with 0.1 mg/kg Env peptides [24] (Pep1|YLRDQQLLGIWG, Pep2|RQQQNNLLRAIEA, Pep3|VYYGVPVWKEA, Pep4|LWDQSLKPCVKLT, Pep5|SVITQACSKVSFE, Pep6|GTGPCTNVSTVQC, Pep7|YKVVKIEPL, GenScript, New Jersey, U.S.) to establish low-level immunity. They thereafter at 11 weeks received 1 mg/kg Env peptides plus 0.1 mg/kg MAB273 s.c. to measure the boost effect. In the prophylactic vaccination group, animals were co-injected with 0.1 mg/kg Env peptides and 0.1 mg/kg MAB273 s.c. with a prime and boost regimen (7 weeks apart) and with 1 mg/kg Env peptides and 0.1 mg/kg MAB273 s.c. as the second boost at 11 weeks and with 1 mg/kg Env peptides, 0.1 mg/kg MAB273 and 100 μg Env protein (426c NFL trimer, provided by Richard Wyatt, Scripps Research) as the third boost at 24 weeks. Blood draws were performed before vaccination and at 48 h, week 1, 2, 3, 7, 8, 9, 11, 12, 13 as well as 48 h after the 2^nd^ boost in heparin tubes. The prophylactic vaccination group was followed for additional 4 weeks. Bronchoalveolar lavage (BAL) sampling was performed as described previously [25] at week 9 and 13.

For the biodistribution assessment, three animals were immunized with 0.1 mg/kg Alexa Fluor 680-labeled MAB273 and terminated after 24 h (one animal) or 48 h (two animals). Multiple samples were collected, such as skin biopsies from the injection site of MAB273, the injection site in the skin on the contralateral leg that received saline, draining left and right inguinal LNs, left and right common iliac LNs, paraaortic LNs, mediastinal LNs, PBMCs, bone marrow, spleen, liver and BAL. The samples were processed into single-cell suspensions as described previously [26–28].

### Blood sample processing

Human and non-human primate (NHP) PBMCs were isolated by Ficoll-Paque (GE Healthcare, Fairfield, CT) density gradient centrifugation of blood samples at 2200 rpm for 25 min with no brake or acceleration as described previously [27, 29]. PBMCs were washed in phosphate-buffered saline (PBS). Samples were either stained or used in in vitro experiments as described or frozen in 90% heat-inactivated fetal bovine serum (FBS) and 10% DMSO (Sigma-Aldrich) for storge in liguid nitrogen tanks for later use.

### Safety, clinical chemistry and hematology tests

For NHP-derived blood samples, hematological analyses of heparinized blood were performed within 8 h after collection on an Exigo Vet instrument (Model H400, Boule Diagnostics AB, Spånga, Sweden) after QC with use of Boule Vet Con control blood. Heparinized plasma samples were analyzed using an ABAXIS Vetscan VS2 3.1.35 Chemistry analyzer (Triolab, Solna, Sweden). Indicated parameters were analyzed on Mammalian Liver Profile rotors (Triolab), which have individual QC controls.

### Assessment of cell fluctuation and phenotype of immune cells

In the in vitro assays, fresh human or NHP PBMCs were first exposed to the monoclonal test antibodies; MAB273, CP-870,893, isotype control IgG1-LALA, Fab/F(ab’)2 fragments of MAB273 or 5 µg/mL TLR7/8L (InvivoGen, tlrl-r848) for 2 h at 4 °C (to assess CD40 binding) or 24 h at 37 °C (to assess cell activation). For both the in vitro and in vivo assays, fresh human and/or NHP PBMCs were stained with LIVE/DEAD™ Fixable Blue Dead Cell Stain Kit (Invitrogen, L23105) at 4 °C for 5 min and subsequently incubated with FcR Blocking Reagent (Miltenyi Biotec, 130-059-901) at 4 °C for 5 min according to manufacturer’s instructions. Samples were then surface stained with a panel of fluorescently labeled antibodies (Table S1). For cell surface staining, the commercial FITC anti-CD40 monoclonal antibody clone 5C3 (1 μg/test) was included in the staining panel and used according to BioLegend’s manual and previously published studies [26, 27]. The 5C3 epitope is known to overlap partly with CP-870,893 and compete with CD40L [30, 31]. After staining and washing, PBMCs were resuspended in 1% paraformaldehyde (PFA) and acquired on an LSRFortessa flow cytometer (Fortessa, BD). Data analysis was performed using FlowJo v10.

**Fig. 1:**
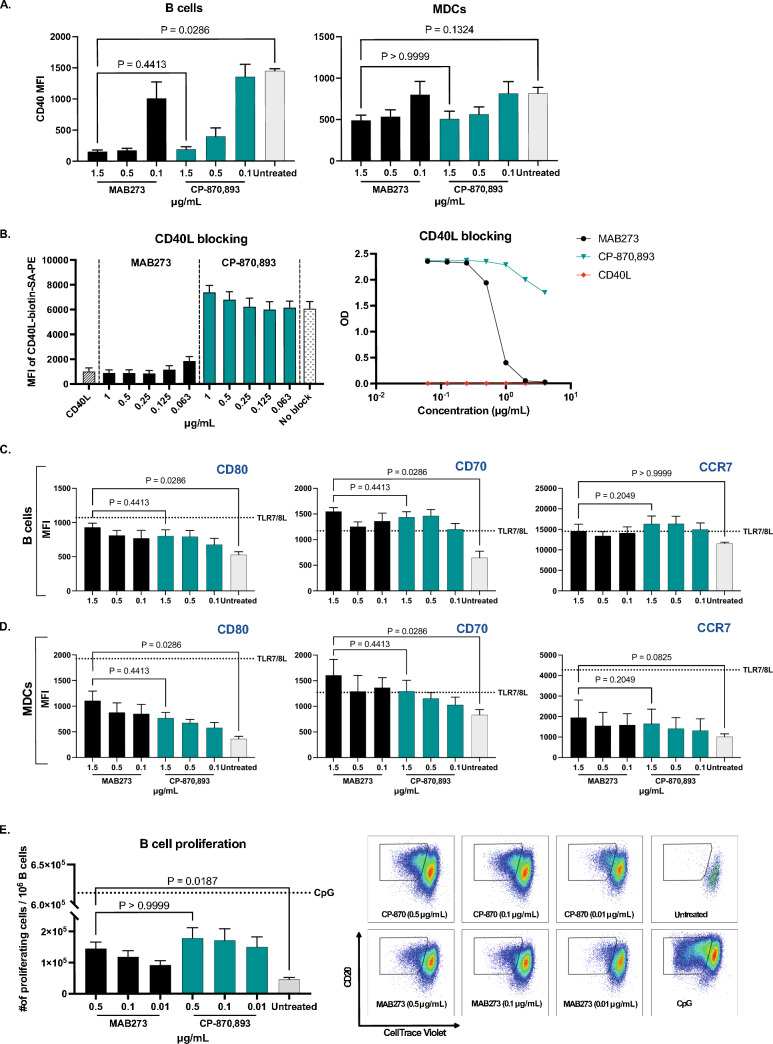
MAB273 binds CD40 and activates human B cells and MDCs with a similar potency as CP-870,893 in vitro. **A** Surface expression levels of CD40 were evaluated by flow cytometry after exposure with MAB273 or CP-870,893. *n* = 3, mean ± SEM. **B** Flow cytometry (left) and ELISA (right) results show the signal of competitive CD40L-biotin-streptavidin conjugate after culture with MAB273 or CP-870,893 or CD40L. *n* = 3, mean ± SEM. **C–D** Human PBMCs were stimulated with MAB273, CP-870,893 or TLR7/8L (positive control). Costimulatory markers CD80, CD70 and LN homing marker CCR7 on B cells (**C**) and MDCs (**D**) were evaluated by flow cytometry. *n* = 3, mean ± SEM. **E** Human PBMCs were stimulated with MAB273, CP-870,893 or CpG (positive control) for 5 days. B cell proliferation is displayed as the number of CellTrace Violet negative B cells per 10^6^ B cells. Representative flow cytometry plots are shown. *n* = 3 + 3, mean ± SEM

**Fig. 2:**
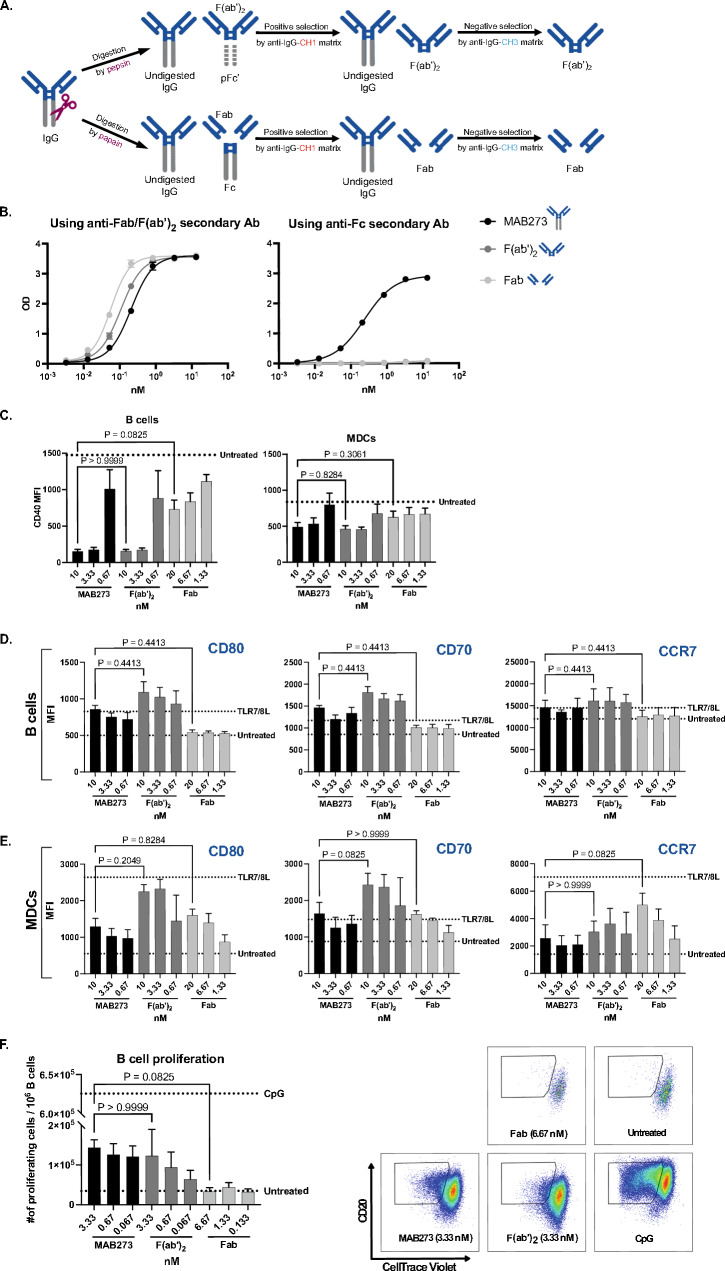
CD40 binding and activation retain the same after removing the Fc region of MAB273. *n* = 3, mean ± SEM. **A** Cartoon shows the process of generating Fab and F(ab’)2 fragments of MAB273. **B** ELISA results show the CD40 binding capacity of complete MAB273, its Fab or F(ab’)2 fragments, the readouts of OD values are displayed by using anti-Fab/F(ab’)2 (left) or anti-Fc (right) secondary antibodies, respectively. **C** Surface expression levels of CD40 on human PBMCs were evaluated by flow cytometry after exposure with complete MAB273, its F(ab’)2 or Fab fragments. The concentrations used were converted to nM for equal comparison. **D–E.** Human PBMCs were stimulated with complete MAB273, F(ab’)2, Fab or TLR7/8L (positive control). Costimulatory markers CD80, CD70 and LN homing marker CCR7 on B cells (**D**) and MDCs (**E**) were evaluated by flow cytometry. **F** Human PBMCs were stimulated with complete MAB273, F(ab’)2, Fab or CpG (positive control) for 5 days. B cell proliferation is displayed as the number of CellTrace Violet negative B cells per 10^6^ B cells. Representative flow cytometry plots are shown

The μg/mL in Fig. 1 was converted to nanomolar concentration (nM = nmol/L) in Fig. 2. The concentrations of MAB273 in Fig. 1 were the same as in Fig. 2. For comparison of the F(ab’)2/Fab fragments and intact MAB273, the same nM of F(ab’)2 and intact MAB273 was used while the nM of Fab was doubled.

### Flow cytometry for labeled antibody tracking

Single-cell suspensions from collected NHP samples were prepared as described previously [26, 28] and stained with LIVE/DEAD™ Fixable Blue Dead Cell Stain Kit (Invitrogen, L23105) prior to FcR Blocking using the FcR Blocking Reagent (Miltenyi Biotec, 130-059-901) according to manufacturer’s instructions. Cells were then surfaced stained with a panel of fluorescently labeled antibodies (Table S1). After staining and washing, cells were spiked with AccuCount beads (Spherotech, ACBP-100-10) and resuspended in 1% PFA and acquired on an LSRFortessa flow cytometer. Counting bead normalized cell numbers were calculated according to the manufacturer’s instruction.

### Detection of antigen-specific T cells

Fresh NHP PBMCs were stimulated with 2 μg/mL Env peptides or 10 μg/mL Env protein overnight at 37 °C, BV421-CD107a staining antibody (BioLegend, 328626) was added during this incubation. Next day, GolgiStop (Monensin, BD, 554724) and Golgi Plug (Brefeldin A, BD, 555029) were added 6 h prior to staining according to manufacturer’s instructions. The LIVE/DEAD staining was performed as described above. Samples were then surfaced stained, permeabilized with Cytofix/Cytoperm^™^ (BD, 554714) according to manufacturer’s instructions, and intracellular staining was performed at 4 °C for 20 min (Table S1). After staining and washing, PBMCs were resuspended in 1% PFA and acquired on an LSRFortessa flow cytometer. The background was subtracted based on the unstimulated autologous controls.

### Analysis of B cell proliferation

Fresh human or NHP PBMCs were labeled with 0.25 µM CellTrace Violet (Invitrogen, C34557) at a cell concentration of 1 million/mL for 20 min at 37 °C. Labeled PBMCs were then incubated with testing antibodies, 1 µg/ml CpG-B (Invivogen, tlrl-2006) or left untreated in complete media (RPMI1640, 10% FBS, 1% L-glutamine, 1% penicillin/streptomycin) and were cultured for 5 days. Some samples were cultured for 5 days under 10 ng/mL IL-4 and 50 ng/mL IL-21 supplemented condition as described earlier [32]. After culture, cells were washed with PBS and stained with LIVE/DEAD prior to FcR Blocking as described above. Samples were then surface stained with a panel of fluorescently labeled antibodies (Table S1). After staining and washing, PBMCs were resuspended in 1% PFA and acquired on an LSRFortessa flow cytometer.

### CD40L competition assay by flow cytometry

Fresh human PBMCs were cultured with different concentrations of MAB273, CP-870,893 or CD40L (ThermoFisher, 34-8902-81) for 20 min at 4 °C and were then washed with cold PBS prior to incubation with 1 μg/mL CD40L-biotin (ThermoFisher, 15836427) for 20 min at 4 °C. Cells were washed with cold PBS and LIVE/DEAD staining as well as cell surface staining with a panel of fluorescently labeled antibodies was performed as described above (Table S1). After staining and washing, PBMCs were resuspended in 1% PFA and acquired on an LSRFortessa flow cytometer.

### ELISA assay for CD40L competition

Greiner-Bio One 96 well half-area ELISA plates (VWR, 738–0032) were coated overnight at 4 °C with 2 µg/ml CD40 protein (ThermoFisher, A42565) in fresh PBS. The plates were blocked with PBS containing 5% (w/v) milk for 1 h at room temperature (RT). Serially diluted MAB273, CP-870,893, or CD40L (2 µg/mL) were added to plates and incubated for 2 h at RT. Then, 2 μg/mL CD40L-biotin was added to the plates and incubated for 1 h at RT. The CD40L-biotin was detected by adding a 1:1000 dilution of Streptavidin-HRP (Mabtech, 3310-9-1000) and the signal was developed by addition of TMB substrate (BioLegend). The addition of an equal volume of 1 M H_2_SO_4_ stopped the reaction, and the optical density (OD) was read at 450 nm and background was read at 550 nm. The plates were washed three times between each incubation step using PBS supplemented with 0.05% Tween 20.

### ELISA assay for CD40 binding

ELISA plates were coated with 2 µg/ml CD40 protein and blocked as described above. Serially diluted MAB273, its Fab or F(ab’)2 fragments were added to plates and incubated for 2 h at RT. The CD40 binding signal was detected by adding a 1:5000 dilution of goat anti-human Fab/F(ab’)2 IgG secondary-HRP antibody (Jackson ImmunoResearch, 109-035-006) or a 1:20,000 dilution of goat anti-human Fc IgG secondary-HRP antibody (Jackson ImmunoResearch, 109-035-008) and the signal was developed as described above.

### MAB273 fluorochrome-labeling

MAB273 was labeled using the Alexa Fluor^™^ 680 Protein Labeling Kit (ThermoFisher, A20172) according to manufacturer’s instructions. The Alexa Fluor 680-conjugated MAB273 was assessed for signal intensity and activation capacity by staining NHP PBMCs overnight. Samples were then washed and acquired on an LSRFortessa flow cytometer. The capacity to bind CD40 was tested by ELISA and compared to non-conjugated MAB273 as described above.

### ELISA assay for detection of anti-MAB273 IgG in NHP plasma

ELISA plates were coated with 1 µg/ml MAB273 and blocked as described above. Serially diluted NHP plasma samples were added to plates and incubated for 2 h at RT. The anti-MAB273 IgG was detected by adding a 1:5000 dilution of anti-macaque pan-species IgG HRP antibody (Absolute Antibody, clone 1B3) and the signal was developed as described above.

### ELISA assay for detection of anti-Env peptides IgG in NHP plasma

ELISA plates were coated with 2 µg/ml NeutrAvidin (ThermoFisher, 31000) and blocked as described above. Then, 2 µg/mL biotin conjugated Env peptides (GenScript, customized) was added to plates and incubated for 1 h at RT. Serially diluted NHP plasma samples were added to plates and incubated for 2 h at RT. The anti-Env peptides IgG was detected by adding a 1:5000 dilution of polyclonal anti-monkey IgG HRP antibody (Nordic MUBio, GAMon/IgG(Fc)/PO) and the signal was developed as described above.

### ELISA assay for detection of anti-Env protein IgG in NHP plasma

ELISA plates were coated with 2 µg/ml mouse anti-His tag antibody (R&D Systems, MAB050) and blocked as described above. Then, 2 µg/mL Env protein (NFL 426c, His-tag) was added to plates and incubated for 1 h at RT. Serially diluted NHP plasma samples were added to plates and incubated for 2 h at RT. The remaining steps are the same as above.

### Analysis of MAB273 pharmacokinetics in NHP plasma

ELISA plates were coated with 2 µg/ml CD40 protein and blocked as described above. Serially diluted NHP plasma was added to plates and incubated for 2 h at RT. MAB273 was detected by adding a 1:5000 dilution of monkey cross-adsorbed polyclonal goat anti-human IgG HRP antibody (Southern Biotech, 2049-05) and the signal was developed as described above.

### Detection of cytokines by ELISA

Human or NHP PBMCs at 1 million /well were stimulated with different conditions in 200 μL per well of a U bottom 96-well plate for 24 h at 37 °C. The supernatant from cell cultures or rhesus plasma samples was evaluated for IL-12 p40 (standard ranges 10–1000 pg/ml), IL-6 (standard ranges 10–1000 pg/ml), IFN-γ (standard ranges 4–400 pg/ml) and TNF (standard ranges 4–400 pg/ml) levels by ELISA using commercially available kits from Mabtech; 3450-1H-6, 3460-1H-6, 3421M-1H-6 and 3512M-1H-6. Assays were performed according to manufacturer’s instructions.

### Bulk transcriptomics

Skin punch biopsy (MAB273 injection site and saline injection site), inguinal lymph nodes (LNs) (MAB273 injection site and saline injection site), and blood (pre-immunization and 24–48 h after immunization) samples were collected from three animals and preserved in RNAlater^™^ Stabilization Solution (ThermoFisher, AM7021) or PAXgene^®^ Blood RNA Tube (BD, 762165). RNA isolation, library preparation, and sequencing were processed at the BEA core facility, Karolinska Institutet, using Illumina stranded mRNA prep kit. Illumina NovaSeq 6000 platform was used to generate paired-end reads of 150 bp with an average sequencing depth of 40 million reads per sample. Samples were preprocessed using nf-core rnaseq pipeline (version 3.7), genome alignment was processed using STAR alignment (version 2.7.10a) to the *Macaca mulatta* genome (Mmul_10) and quantification with Salmon (version 1.8.0).

### RNA sequencing data analysis

For this study, we used a customized bioinformatic analysis workflow of RNA sequencing data using R (version 4.1.2). Differential gene expression analysis was performed using DESeq2 (version 1.34.0). Gene Set Enrichment analysis was done with ClusterProfiler (version 4.2.2) package. The database used for gene set enrichment analysis was the Blood Transcriptome Modules (BTMs) [33]. To compare differentially expressed genes, Wald test was performed with multiple hypothesis testing controlling the false discovery rate (FDR) using the Benjamini–Hochberg procedure (*q* value < 0.05).

### Statistics

A Friedman test was used when three or more groups were compared; the results were considered statistically significant when *p* < 0.05, and the *p* values are shown in the graphs. Analyses were performed in GraphPad Prism 9.

## Results

### MAB273 binds the CD40L binding site and activates immune cells

Extensive screening of novel CD40 binding antibodies after immunization of rabbits with human recombinant CD40 and isolation of B cell clones resulted in the identification and subsequent production of MAB273 engineered with human IgG1 backbone containing the L234A and L235A (LALA) mutations as recently described [23]. In this study, we focused on further functional characterization of MAB273. Since CP-870,893 is one of the most well-studied and potent CD40 antibodies in clinical development we used it as a comparator in the in vitro assays. CD40 binding capacity and activation were analyzed on multiple immune cells but we focused on B cells and myeloid dendritic cells (MDCs) within the PBMC population since they are central in immunity and express high levels of CD40 (Figure S1A). Human PBMCs exposed to MAB273 or CP-870,893 were found to have markedly reduced signal of a CD40 staining antibody (clone: 5C3) confirming that they both compete for binding to CD40 in a dose-dependent manner (Fig. 1A). However, using a CD40L competition assay only MAB273 binding was affected confirming that the epitope specificity on CD40 is different between the two antibodies and only MAB273 binds the CD40L binding site while CP-870,893 is known to bind outside [34] (Fig. 1B). Regardless, both B cells and MDCs showed that they had upregulated the costimulatory markers CD80, CD70 and LN homing marker CCR7 after MAB273 or CP-870,893 exposure (Fig. 1C and D). B cell proliferation, as assessed by dilution of CellTrace violet dye, also showed similar activation with MAB273 or CP-870,893 stimulation (Fig. 1E). Although induction of phenotypic differentiation and cell proliferation was clear with both MAB273 and CP-870,893, we did not find detectable cytokines such as IL-12 p40, IL-6, IFN-γ and TNF in the cell culture supernatants after stimulation (data not shown).

### CD40 binding and activation capacities retain after removing the Fc region of MAB273

In order to evaluate if MAB273 is FcγR-independent and whether avidity plays a role, we generated F(ab’)2 fragments by pepsin digestion to cleave off the Fc region but maintain the hinge region as well as Fab fragments by papain digestion to remove both the Fc region and hinge region (Fig. 2A). The purity, correct valency and low endotoxin content of those fragments were verified before their use in the in vitro cultures (Figures S1D and S1E). The Fab and F(ab’)2 fragments of MAB273 still bound to CD40 but were not detected by an anti-Fc antibody (Fig. 2B). Human B cells and MDCs exposed to MAB273 or the F(ab’)2 fragment showed similar ability to block the CD40 staining antibody in a dose dependent manner, while the Fab fragment showed weaker CD40 blocking capacity (Fig. 2C). In addition, MAB273 and the F(ab’)2 exposure resulted in similar upregulation of CD80, CD70 and CCR7 on B cells (Fig. 2D) and MDCs (Fig. 2E). The Fab fragment retained the activation capacity on MDCs (Fig. 2E) but was weaker for B cells (Fig. 2D) suggesting that B cells may require higher avidity for activation (Figure S1A). In addition, B cell proliferation induced by MAB273 or F(ab’)2 was similar but Fab showed weaker induction (Fig. 2F). This demonstrates that the F(ab’)2 fragment of MAB273 has retained immunostimulatory capacities in vitro after removing the Fc region, but the Fab fragment is less potent for B cell stimulation. We concluded that MAB273-induced activation is not dependent on FcγR crosslinking.

### MAB273 binds CD40 and activates rhesus macaque PBMCs in vitro

**Fig. 3:**
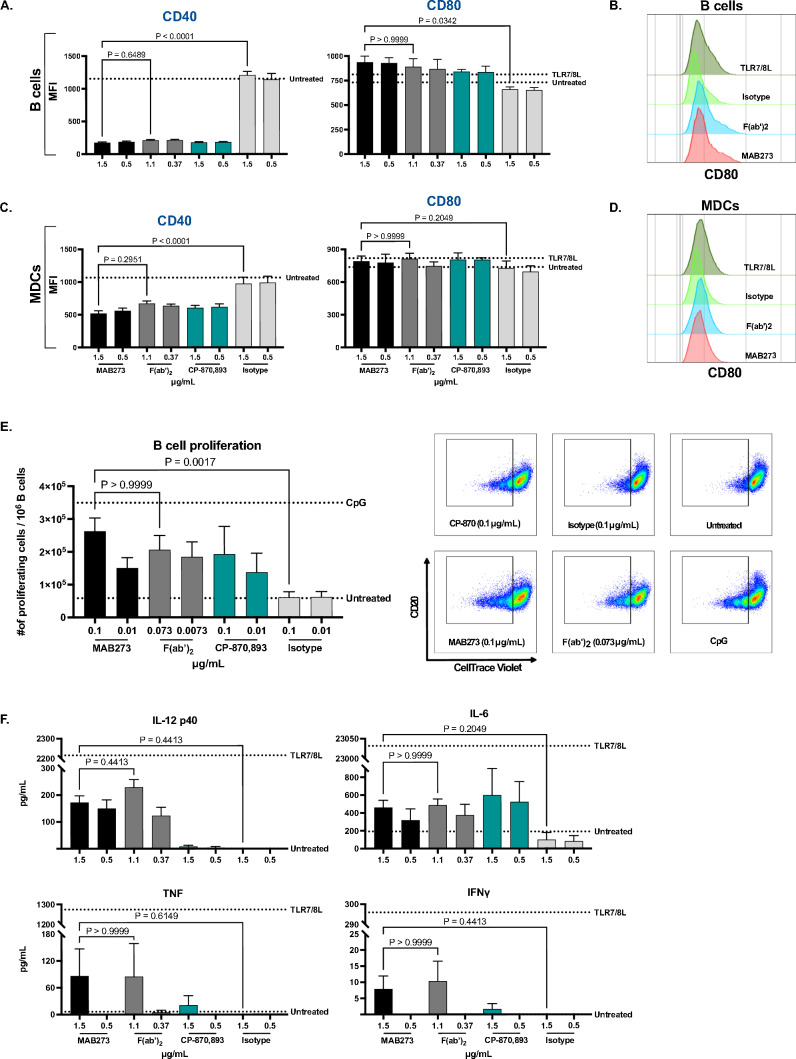
MAB273 shows potent CD40 binding and activation capacities in rhesus macaque PBMCs in vitro. Rhesus PBMCs were stimulated with MAB273, CP-870,893, isotype control antibody, the F(ab’)2 fragment of MAB273 (similar molar concentrations to complete MAB273) or TLR7/8L (positive control). **A–D** Surface expression levels of CD40 and costimulatory marker CD80 on B cells (**A**) and MDCs (**C**) were evaluated by flow cytometry. *n* = 3, mean ± SEM. Representative flow cytometry histograms of CD80 on B cells (**B**) and MDCs (**D**) are shown. **E** Rhesus PBMCs were stimulated with MAB273, CP-870,893, isotype control antibody, the F(ab’)2 fragment of MAB273 (similar molar concentrations to complete MAB273) or CpG (positive control) for 5 days. B cell proliferation is displayed as the number of CellTrace Violet negative B cells per 10^6^ B cells. Representative flow cytometry plots are shown. *n* = 3 + 3, mean ± SEM. **F** Levels of cytokines (IL-12 p40, IL-6, TNF and IFN-γ) were measured by ELISA, supernatants used were taken from (**A**) and (**C**). *n* = 3, mean ± SEM

With the aim to utilize a physiological in vivo animal model, we next tested the ability of MAB273 to bind and stimulate rhesus macaque PBMCs in vitro by repeating many of the above in vitro experiments. The immune cells expressed CD40 as expected where B cells and MDCs had the highest expression and T cells and neutrophils low expression (Figure S1B). Phenotypic differentiation after stimulation with MAB273, its F(ab’)2 fragment, CP-870,893 or the isotype control antibody (IgG1-LALA) were analyzed. Again, the signal of the CD40 staining antibody was blocked when the cells had been exposed to MAB273, F(ab’)2 or CP-870,893 but not to the isotype control (Fig. 3A and C). In addition, upregulation of CD80 was found on rhesus cells after stimulation with either by MAB273, F(ab’)2 or CP-870,893, although it was less pronounced compared to observed in human cells (Fig. 3A–D). No upregulation was found by the isotype control antibody. B cell proliferation was also induced by MAB273, F(ab’)2 or CP-870,893 exposure but not by the isotype control antibody (Fig. 3E). MAB273 and F(ab’)2 induced low but detectable levels of IL-12 p40, IL-6, TNF and IFN-γ secretion (Fig. 3F), while only low levels of IL-6 was induced by CP-870,893. This shows that MAB273 can bind to rhesus macaque CD40 and activate immune cells. As in human cells, the F(ab’)2 fragment of MAB273 has retained immunostimulatory capacities in rhesus cells.

### MAB273 induces innate immune activity in vivo in rhesus macaques

**Fig. 4:**
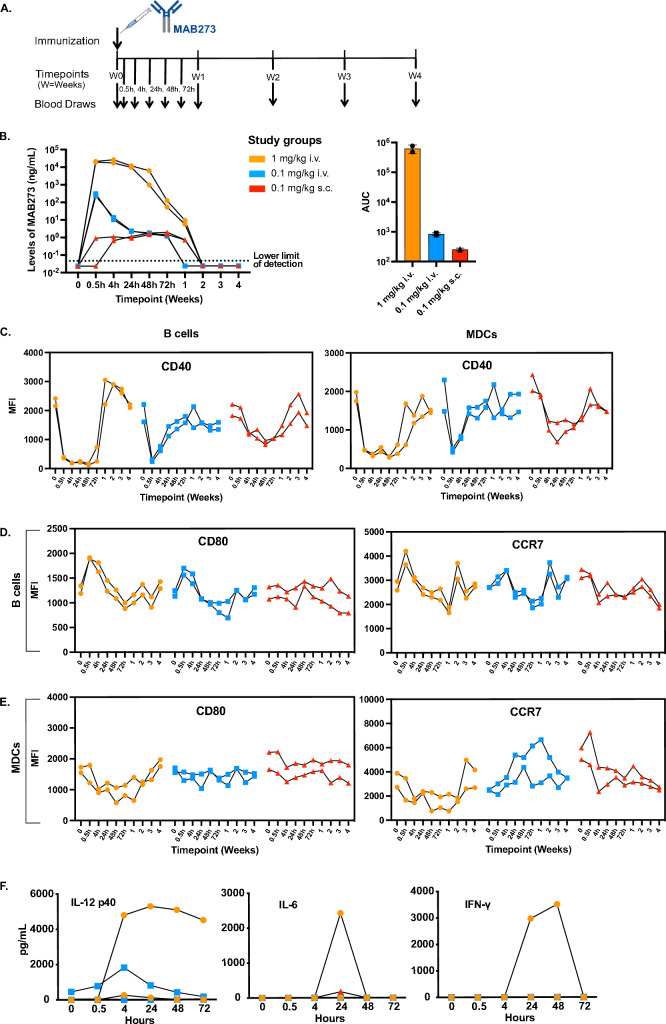
In vivo innate immune activity in rhesus macaques. **A** Outline for toxicity and safety study in rhesus macaques administered 1 mg/kg i.v., 0.1 mg/kg i.v., or 0.1 mg/kg s.c. (*n* = 2 per group). **B** Levels of MAB273 in plasma over time (left), AUC (area under curve, right) is calculated after normalizing (left) to linear axes. *n* = 2 per group. **C–E** Surface expression levels of CD40 (**C**), costimulatory marker CD80 and LN homing marker CCR7 on B cells (**D**) and MDCs (**E**) were evaluated by flow cytometry over time. *n* = 2 per group. **F** Systemic levels of pro-inflammatory cytokines (IL-12 p40, IL-6, IFN-γ) in plasma over time. *n* = 2 per group

Six rhesus macaques were thereafter divided into three groups to receive MAB273 administration at different doses and routes (Fig. 4A). Standard clinical chemistry analyses, including a series of liver and kidney function and complete blood count (CBC) measurements were performed in addition to immunological analyses. The first group that received the highest dose of 1 mg/kg by intravenous (i.v.) administration showed that several clinical chemistry parameters including alkaline phosphatase (ALP), alanine transaminase (ALT), gamma-glutamyl transferase (GGT), bile acid (BA), total bilirubin (TBIL) and blood urea nitrogen (BUN) were elevated above the normal reference range (Figure S2A). This group also showed side effects characterized by loss of appetite and reduced activity behavior for up to 5 days. In contrast, the lower dose of 0.1 mg/kg given i.v. did not induce any detectable side effects and most of the clinical chemistry parameters remained within the healthy range (Figure S2A). The low dose was thereafter tested with subcutaneous (s.c.) administration which also did not induce side effects. As visualized by CBC, there were fluctuations of cell numbers following administration of MAB273 found with both doses and routes. A rapid decline in platelets was observed already at 0.5–4 h accompanied by a rapid increase in white blood cell counts, especially granulocytes, after MAB273 administration in all groups (Figure S2B). Frequencies of specific cell subsets identified by flow cytometry and normalized to the CBC data confirmed a rapid increase of neutrophils while there was a transient decline of both B cells and MDCs. The cell fluctuations were dose-dependent and with a notably more dramatic effect in the 1 mg/kg i.v. group (Figure S2C). The transient fluctuation of immune cells after MAB273 administration may stem from redistribution of activated cells leaving the circulation to migrate to tissues followed by a replenishment of cells from the bone marrow as has been proposed earlier [11, 22]. Body weight remained stable during the entire study period in all groups (Figure S2D).

Analysis of the pharmacokinetics (PK) of MAB273 in plasma showed that the levels were readily detectable after 0.5 h of administration in both of the i.v. groups (Fig. 4B). In the high dose group, the levels of MAB273 peaked around 0.5–4 h and then declined gradually until it was undetectable after 2 weeks. In the low dose group, the highest level was detected at 0.5 h, then continually decreased and was undetectable after 1 week. In the s.c. group, MAB273 was detectable at 0.5–4 h but at 2–4 log lower levels compared to the i.v. groups. However, the level of MAB273 in the s.c. group was sustained for a week and undetectable at 2 weeks. This suggests that s.c. administration results in a depot effect with slower release of MAB273 into the circulation.

Binding of MAB273 to CD40 in vivo was evaluated by quantifying the loss of detection signal from the staining CD40 antibody as performed in the in vitro experiments. Rapidly (0.5 h) after administration of MAB273, detection of CD40 was blocked on B cells and MDCs (Fig. 4C). Lack of CD40 signal was sustained for 72 h in the high-dose i.v. group while this was found for a shorter period for the low-dose i.v. group. The s.c. group also showed reduced signal for CD40, but this was noticed later (at 4 h) and sustained for 2 weeks in line with the observed pharmacokinetics of MAB273 in plasma. As mentioned above, the return of detectable CD40 expression may be due to replenishment of new cells into the circulation as well as the half-life of MAB273. Accompanied with MAB273 binding to CD40 on immune cells, a rapid increase in CD80 and CCR7 expression was observed, especially in i.v. groups (Fig. 4D). The expression gradually returned to baseline levels or even below which may be explained by that newly recruited cells exhibit a more immature phenotype. The upregulation of CD80 and CCR7 on MDCs was less noticeable than on B cells (Fig. 4E). Secretion of IL-12 p40, IL-6 and IFN-γ was detected in one of the animals receiving the high dose while most animals did not show detectable levels (Fig. 4F). TNF was not detected (data not shown). In conclusion, MAB273 induces strong innate immune activation with regard to cell recruitment and activation while being well tolerated at the dose of 0.1 mg/kg given either i.v. or s.c. in rhesus macaques. Since s.c. administration demonstrated clear immune stimulation while potentially offering a depot effect of MAB273 for slower release and better tolerability, this route may be more attractive for clinical development, and hence this was used in our subsequent studies.

### MAB273 targets and activates immune cells at the site of injection and draining LNs

**Fig. 5:**
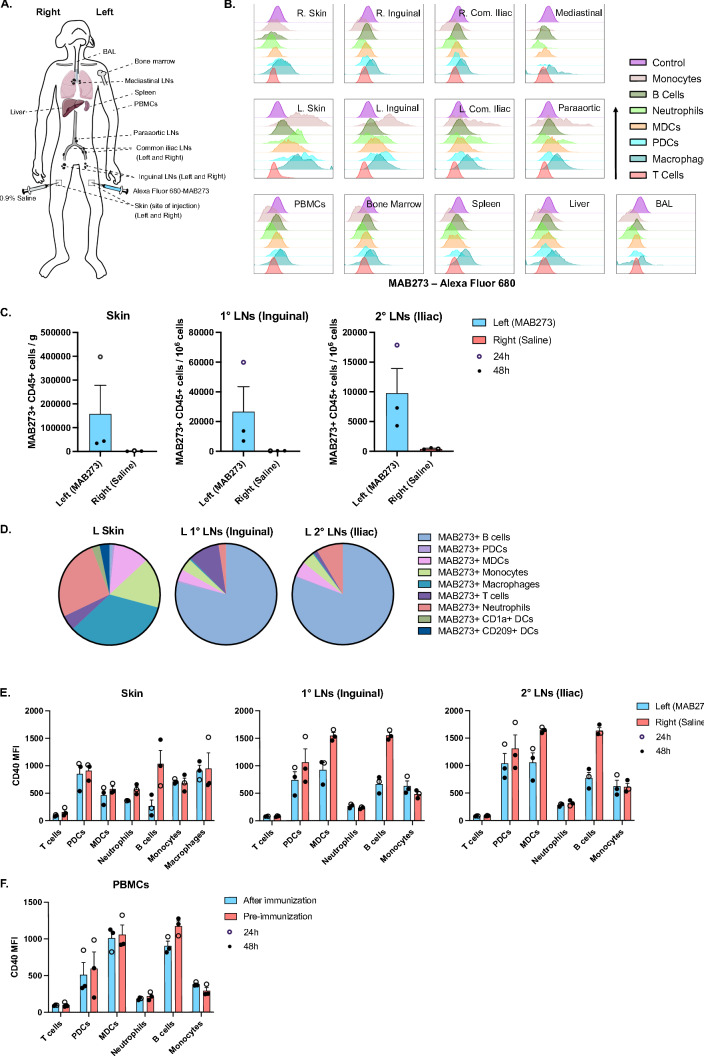
In vivo biodistribution of MAB273. **A** Rhesus macaques (*n* = 3) were administered 0.1 mg/kg s.c. of Alexa Fluor 680-MAB273 in the skin above the left quad and 0.9% saline solution s.c. in the skin above the right quad. The cartoon shows the sites of immunization and sampling performed after 24 h (*n* = 1) or 48 h (*n* = 2). **B** Histograms show Alexa Fluor 680-MAB273 signal on different cell populations in different tissues of one representative animal. Control = peripheral blood B cells from the same animal before immunization with labeled MAB273. **C** MAB273 + CD45 + cells normalized by counting beads at site of injection or draining LNs. *n* = 3, mean ± SEM. Compiled graphs show one animal that was sampled at 24 h (open circle), and two animals that were sampled at 48 h (closed circle). **D** Pie charts show proportion of different CD45 + immune cells targeted with MAB273 at the injection site, the primary and secondary draining LNs. The values are the average of three animals. **E–F** Expression of CD40 at site of injection and draining LNs (**E**) as well as PBMCs (**F**). Compiled data were evaluated by flow cytometry. Geometric mean fluorescence intensity (MFI) is shown. *n* = 3, mean ± SEM

To understand the biodistribution of MAB273 in different tissues after administration, the antibody was labeled with AlexaFluor 680 fluorochrome to enable tracking in vivo. The fluorescent signal and unaltered CD40 binding and activation capacities of the labeled MAB273 were validated in vitro before administered in vivo (Figures S3A–C). Three animals were immunized s.c. and biopsies were collected after 24 (*n* = 1) or 48 h (*n* = 2) from the sites of injection, LNs and other selected tissues (Fig. 5A). MAB273-AlexaFluor 680 was predominantly detected at the site of injection (skin of the left thigh) and the LNs specifically draining this site (left inguinal LNs, left common iliac LNs and paraaortic LNs). Monocytes, B cells, neutrophils, MDCs, PDCs and macrophages showed detectable MAB273 binding while T cells had no signal (Fig. 5B). MAB273 signal was not detected at the saline control injection site in the skin of the opposite thigh (right) and the LNs draining this site (right inguinal LNs and right common iliac LNs). No or very weak signal was detected in draining peripheral tissues such as the liver, spleen, bone marrow, BAL and PBMCs (Figs. 5B and S4A). There were more CD45 + immune cells targeted with MAB273 at the injection site and the primary draining LNs compared to the secondary draining LNs more distant from the injection site (Fig. 5C). In the skin, the most abundant cell subsets targeted with MAB273 were macrophages, neutrophils and monocytes likely due to CD40 expression and phagocytic ability [35–37]. In the draining LNs, B cells were predominantly targeted with MAB273 likely due to that they represent a major CD40 expressing population [38–40] (Fig. 5D). In line with the detectable signal of MAB273, there was considerable infiltration of immune cells to the injection site in the skin compared to the saline-injection sites. This consisted of mainly infiltrating B cells, MDCs, monocytes, neutrophils and T cells (Figures S4B and S4C). Again, reduced CD40 staining signal was observed at the injection site, draining LNs and PBMCs indicative of MAB273 binding to CD40 expressing cells as expected (Fig. 5E and F). We therefore concluded that MAB273 has restricted biodistribution to the site of injection in the skin and the specific draining LNs and that multiple cell subsets are targeted but to the highest degree B cells, DCs and macrophages.

### Strong induction of genes associated with innate immune stimulation in MAB273 targeted tissues

**Fig. 6:**
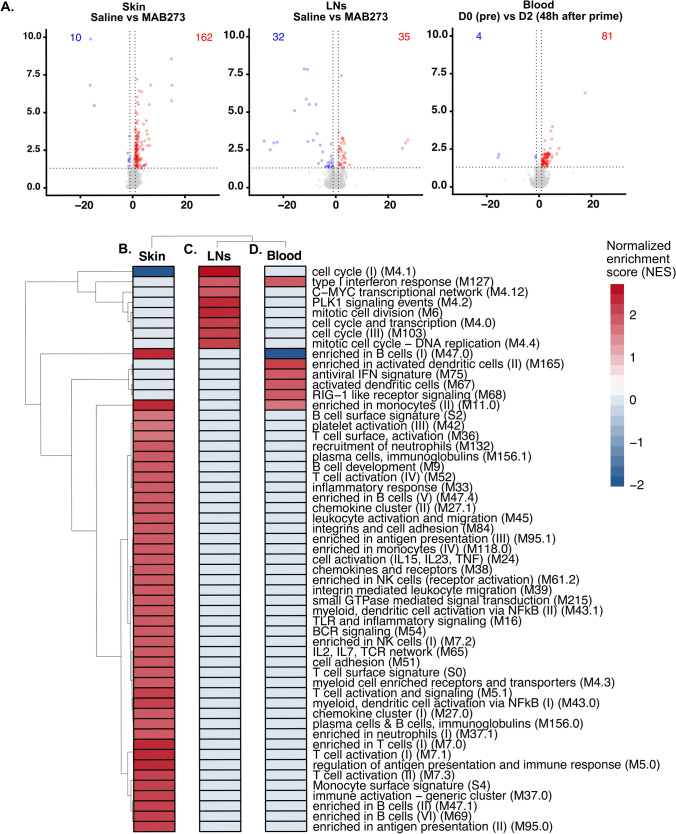
RNA-seq data analysis in different tissues. RNA-sequencing was performed on samples from rhesus macaques administered with AF680-MAB273 (see Fig. 5). *n* = 3. **A** Volcano plots of differentially expressed genes in skin, inguinal LNs and blood. Up-regulated genes are in red and down-regulated genes are in blue. The calculation of differentially expressed genes was based on a control reference for each tissue: D0 pre-immunization (blood) or saline site of injection (LNs and skin). Dotted grey lines indicate fold change > 1 and adjusted *p* values < 0.05. **B–D** Gene seat enrichment analysis (GSEA) of blood transcription modules significantly enriched in skin (**B**), LNs (**C**) and blood (**D**) compared to their respective control samples, color gradient is based on normalized gene set enrichment scores. All statistical comparisons were adjusted by the Benjamini–Hochberg procedure, adjusted *p* values < 0.05 were considered significant. “D0” is the day before prime immunization, “D2” is 48 h after prime

To further understand the immune activation profile induced by MAB273 administration in vivo*,* we performed RNA sequencing analyses on the draining LNs and skin biopsies from the site of injection as well as the blood (Fig. 6A). This revealed a significant number of differentially expressed genes (DEGs) in the MAB273-targeted skin and LNs compared to the donor-matched saline control sites (Fig. 6B and C). In addition, blood taken before MAB273 administration compared to 24–48 h after showed significant gene modulation (Fig. 6D).

Gene set enrichment analysis using the blood transcription modules (BTMs) described previously [33] demonstrated that distinctly different gene modules were changed at the different anatomical sites. The skin had the highest activation and transcriptional changes after MAB273 injection. The upregulated genes in skin included sets of genes associated with specific cell surface markers (*CD19*, *CD2, IL21R)* and chemokines such as the *CXCR5* gene. All the significantly enriched gene modules were upregulated, except the cell cycle modules. The results indicated activation and recruitment of T cells, B cells, NK cells, monocytes, and DCs to the site of injection (Fig. 6B). MAB273-draining LNs also showed that there were genes upregulated compared to the saline-draining LNs. These genes were fewer and were distinct from those observed in the skin. The upregulated genes in the LNs were mainly linked to antigen presentation (*IRAG2*) and interferon (*IRF6*), and a few downregulated genes were linked to RNA processing (*U2*, *U3*, *U4*, *RNaseP*). The enrichment analysis indicated an upregulation of modules related to cell proliferation (mitotic cell division and cell cycle modules) (Fig. 6C). Furthermore, in the blood, the genes differentially expressed 24–48 h after MAB273 administration were mainly associated with interferon signatures, such as *ISG15*, *RSAD2*, and *SKIV2L* as well as genes associated with monocytes and DC activation (Fig. 6D). MAB273 therefore induces significant innate immune activation characterized by monocyte and DC activation in the blood, recruitment of immune cells to the site of injection while cell proliferation and antigen presentation processes were more dominant in the draining LNs.

### MAB273 exhibits adjuvant effects for induction of antigen-specific CD4 and CD8 T cells

**Fig. 7:**
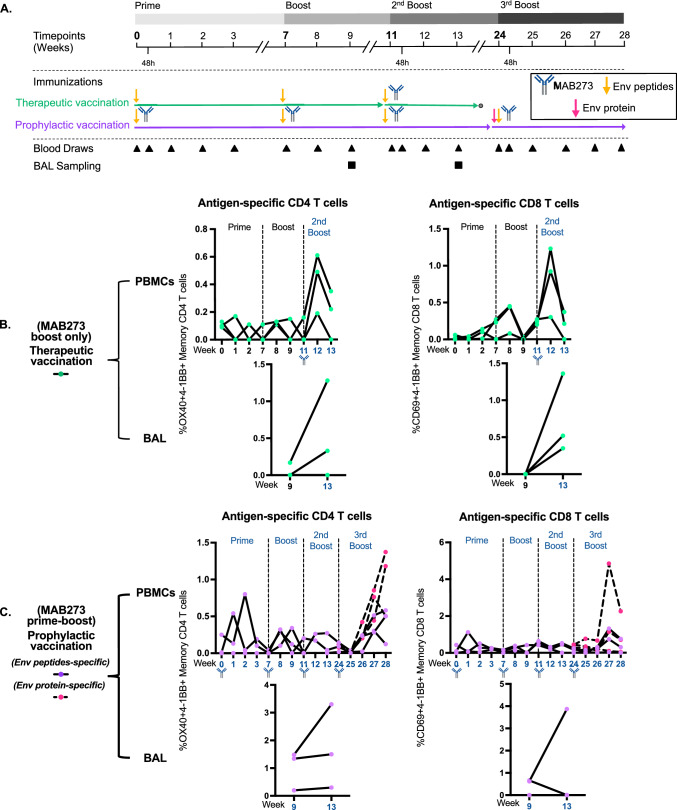
MAB273 can potentiate induction of antigen-specific CD4 T cells and CD8 T cells in PBMCs and BAL. **A** In the therapeutic vaccination group, rhesus macaques (*n* = 3) were administered 0.1 mg/kg s.c. of Env peptides for the first two immunizations then 1 mg/kg Env peptides plus 0.1 mg/kg MAB273 s.c. for the last immunization; in the prophylactic vaccination group, rhesus macaques (*n* = 3) were co-injected with 0.1 mg/kg Env peptides plus 0.1 mg/kg MAB273 s.c. for the first two immunizations then 1 mg/kg Env peptides plus 0.1 mg/kg MAB273 s.c. for the third immunization, followed 1 mg/kg Env peptides, 0.1 mg/kg MAB273 plus 100 μg Env protein for the last immunization. **B** Antigen-specific CD4 T cells and CD8 T cells in PBMCs and BAL in therapeutic vaccination group. *n* = 3. **C** Antigen-specific CD4 T cells and CD8 T cells in PBMCs and BAL in prophylactic vaccination group. *n* = 3

Finally, we evaluated the effect of MAB273 to act as an adjuvant both for therapeutic vaccination where low degree of immunity already exists and also to enhance primary immune responses as in prophylactic vaccination. Three animals therefore first received seven well characterized HIV-1 envelope glycoprotein (Env) peptides [24] as model antigen alone two times to establish low levels of immunity before receiving boost immunizations with MAB273 co-administered s.c. to mimic a therapeutic vaccination (Fig. 7A). In a separate group, three animals received MAB273 together with the Env peptides in a prime-boost schedule of four immunizations to mimic prophylactic vaccination. The final immunization was performed with an additional recombinant trimer Env protein (Fig. 7A).

Low frequencies of Env-specific T cell responses were induced by Env peptide immunization alone (Fig. 7B). The responses were enhanced in two out of three animals when they received a boost with Env peptides and MAB273. This effect was evident for both systemic Env-specific CD4 and CD8 T cells in blood (Fig. 7B) as well as in bronchoalveolar lavage (BAL) (Fig. 7B). Two out of the three animals immunized with Env peptides in combination with MAB273 already at prime immunization induced higher levels of Env-specific CD4 and CD8 T cell responses compared to the animals receiving Env peptides only (Fig. 7B and C). Although the subsequent boost immunizations re-activated T cell responses, they did not reach the peak levels found after the prime immunization (Fig. 7C). This was observed both in blood and BAL and may be a consequence of the low dose of Env peptides (0.1 mg/kg) and the induction of antibodies against the humanized MAB273 in rhesus macaques (Figures S5A and S5B).

The activation profile of MAB273 based on the RNA sequencing and blood transcriptome analysis comparing the activation at pre-immunization compared to the second boost showed that the differences were negligible indicating that recurrent MAB273 administration may result in lower innate immune activation (Figure S5C). Nevertheless, reactivation of memory T cell responses to peak levels occurred after the fourth immunization of MAB273 when using trimer Env protein in combination with Env peptides to provide more antigen (Fig. 7C). No detectable IgG to Env peptides was found (Figure S5D) but IgG to Env protein was detected (Figure S5E). Taken together, our study demonstrates that MAB273 is a potent agonistic anti-CD40 antibody with rapid binding and activation to B cells and MDCs in vitro and in vivo in rhesus macaques and can help enhance antigen-specific T cell responses.

## Discussion

This study provides evidence that MAB273 binds to CD40 and activates human and rhesus macaque B cells and MDCs in vitro and in rhesus macaques in vivo*.* In particular, MAB273 activates CD40 signaling to upregulate T cell costimulatory receptors CD80 and CD70 and LN homing receptor CCR7 on APCs which aids in driving T cell responses as shown by induction of both systemic (blood) and tissue (BAL) immunity. This is in line with what has been reported with other potent anti-CD40 agonistic antibodies [11, 22, 26, 29, 34, 41, 42]. Since no direct in vivo comparison between MAB273 and other anti-CD40 antibodies (e.g., CP-870,893) has been performed yet, any superior adjuvant effect by MAB273 remains elusive. However, MAB273 binds to the CD40L binding site of CD40 and exerts its biological activity independent of FcγR crosslinking which is a unique combined feature that offers novel ways of inducing innate responses.

Structure analysis has demonstrated that a symmetric complex between trimeric CD40L and dimers of CD40 is formed when they interact [43, 44]. CD40 signaling requires large clusters of these complexes [45, 46]. However, CD40L has also been reported to interact with several integrins independent of CD40-CD40L interaction [47, 48]. In fact, multiple CD40L binding sites for integrins may form even larger (anti-CD40 antibody)-CD40-CD40L-intergrin complexes that induce additional biological activities than the canonical CD40 activation which could enhance unwanted side effects [49–53]. Based on this, we screened and developed a series of antibodies, including MAB273, which can effectively replace CD40L and potentially avoid interference by additional CD40-CD40L-intergrin complex formation [23]. Our results confirmed that MAB273 binds to the CD40L binding site. Earlier studies have shown that APX005M, a monoclonal antibody competing with the CD40L binding site, is highly agonistic and may activate CD40 similarly to endogenous CD40L [22, 54].

CD40 signaling induced by agonistic anti-CD40 antibodies has been shown to depend on cells expressing Fc-receptors [18]. In this regard, engineering the Fc region of agonistic CD40 antibodies to promote the FcγR crosslinking can enhance their potency [18–22], but increased crosslinking also augments adverse events [20, 21]. However, agonistic anti-CD40 antibodies of the IgG2 isotype show Fc-independent activity [29, 55]. This is likely provided by conformational regulation and flexibility of disulfide bonds in the hinge region [55, 56] which facilitates CD40 clustering [45, 46] in contrast to the IgG1 isotype which has an unfixed hinge region and highly flexible Fab arms. However, there are also opposite results showing that the activity of IgG2 isotype antibody is not Fc-independent [20]. Taken together, at least in terms of the IgG1 isotype, functional Fc region and FcγR crosslinking are considered necessary for strong CD40 agonists. However, our results with MAB273 demonstrate, both in vitro and in vivo, that it is possible for agonistic CD40 antibodies to be of the IgG1 isotype and function independently of FcγR crosslinking [16]. We have earlier found that the Fc-silenced MAB273 induced more potent immune activity than several variants of CP-870,893, including the strongest Fc-enhanced crosslinking antibody CP-870,893 IgG1-V11 [23], which supports the notion that epitope binding site is critical. Moreover, in this study we observed that only bivalent F(ab’)2, and not monovalent Fab, showed similar agonistic activity as complete MAB273. This suggests that clustering of CD40 by bivalent Fab arms is a critical component of activation and may explain why IgG2 agonists retain activity without Fc engagement [34, 45, 55]. However, further studies such as assessment of aggregation of the Fab fragment to rule out any effects caused by that need to be performed to solidify the findings. Various published agonistic CD40 antibodies show different or even completely opposite functions in terms of CD40L-binding site specificity and FcγR crosslinking [11, 16, 22, 29, 42].

We tested MAB273 in rhesus macaques in order to mimic the human immune system as closely as possible. This was partly driven by that agonistic CD40 antibodies have distinct characteristics in mice and humans [16] and human FcγRs are different from mouse FcγRs [57]. Previous studies have mostly used human CD40 transgenic (hCD40Tg) mice [42, 58], hCD40Tg FcγRIIb-/- mice [55], hCD40Tg FcγR-/- mice [46] or hCD40Tg/mFcgr2b-/-/hFcgr2b ± mice [45]. However, it has been proposed that only humanized CD40/FcγR mice can provide the correct in vivo environment for evaluating human CD40 antibodies [20]. On the other hand, despite that macaque FcγRIIb binds poorly to human antibodies [57], NHP is the preferred preclinical model [22, 26, 27, 29] due to their immunophenotypic similarity and CD40 homology to humans. To this end, a preclinical study of the Fc-unmodified IgG2 anti-CD40 monoclonal antibody CDX-1140 showed that the agonistic activity is Fc-independent and well-tolerated in NHPs [29]. However, the Fc region of CDX-1140 is still functional and thus it is possible that NHPs do not fully predict the activity, toxicity, or Fc-independence in humans. Since MAB273 has double LALA (L234A and L235A)-mutations to completely eliminate Fc-FcγR binding, the NHP model should largely reflect the activity in humans in this regard. This is also supported by that CD40-binding and activation of intact MAB273 and F(ab)’2 fragments showed similar in vitro results in human and rhesus cells. Previous studies demonstrated that LALA critically reduces the binding of the Fc region to all known human FcγRs and the complement component 1q (C1q). As a result, antibody-dependent cellular cytotoxicity (ADCC) and complement-dependent cytotoxicity (CDC) are not induced [59–61]. LALA has no effect on serum clearance [62], nor on PK [63].

Our dose escalation results showed that 1 mg/kg i.v. gave side effects by transient elevation of liver transaminases and behavioral changes like reduced appetite and physical activity, while 0.1 mg/kg did not but still induced robust immune activation. The peak of liver transaminases appeared on day 7 and normalized by day 21 which is delayed compared to results reported for CP-870,893, which appeared between day 2 and 8 in humans [11]. Still, MAB273 induced transient liver abnormality similar to reported by other agonistic CD40 antibodies [11, 12, 21, 26] which may be caused by apoptosis of CD40 expressing hepatocytes and CD40-mediated hyperactivation [11, 64]. However, hepatotoxicity can often be controlled by dose and route of administration as demonstrated with other well-characterized anti-CD40 antibodies such as CP-870,893 and ADC-1013 [12, 21, 65]. This is in line with our results showing that the dose of 0.1 mg/kg of MAB273 did not cause changes in liver function. We observed that the dissemination of fluorescently labeled MAB273 after s.c. administration was predominantly at the site of injection and specific draining LNs and that there was no or only weak signal systemically in the blood. This is similar to our previous study of biodistribution after s.c. administration of another CD40 antibody (341G2) [27]. Also, in this study we found almost no antibody disseminating to the liver after s.c. administration. We have earlier shown that i.v. administration of 341G2 resulted in dissemination to the liver [26, 27], which would likely also be the case for MAB273 with this route. It remains to be tested whether the higher dose of 1 mg/kg MAB273 given s.c. would be better tolerated than this dose given i.v.. Therefore, at this stage we cannot conclude that MAB273 induces less toxicity than other agonistic anti-CD40 antibodies. The transient increase of blood urea nitrogen (BUN) in 1 mg/kg i.v. group could be explained by the difficulty of renal excretion induced by macromolecular drugs (such as antibodies) and consequential renal inflammatory responses [66]. Transient hematologic changes were also observed after administration and aligned well with the pharmacokinetics of MAB273 in all groups in our study. In particular, the number of B cells and MDCs decreased in blood. Also, a rapid decline in platelets at 0.5 h was found, likely caused by activation of CD40-expressing platelets and their contribution to inflammation and aggregation [47, 67]. Our in vitro and RNA sequencing results also suggested that MAB273 can induce B cell proliferation similarly to other studies [26, 29, 42]. The downregulation of the B cell enrichment module in the blood and concomitant upregulation in the skin followed the change in cell numbers and suggest an extravasation and replenishment of immune cells induced by CD40 activation, as has been proposed in previous studies with administration of several different agonistic anti-CD40 antibodies, such as CP-870,893 [11], APX005M [22], CDX-1140 [29] and 341G2 [26, 45]. The rapid increase in numbers of granulocytes, especially neutrophils, at 0.5–4 h likely contributed to most of the observed enrichment in inflammation signatures. The 1 mg/kg i.v. group showed overall higher magnitude and longer duration of inflammation but 0.1 mg/kg given s.c. induced larger fluctuations in cell numbers in blood than 0.1 mg/kg i.v.. This may be caused by that s.c. administration also stimulated cells locally in the skin which resulted in more redistribution of neutrophils [68]. We found that i.v. administration and high dose of MAB273 only can induce detectable systemic cytokine secretion. Cytokines induced by known agonistic anti-CD40 antibodies include IL-12 p40, IL-6, TNF and IFN-γ [11, 26, 27, 29] although the levels vary between individuals and between humans and NHPs [11, 22, 29].

Anti-drug antibodies (ADA), such as the anti-MAB273 IgG observed in our study, are antibodies raised to the administered human antibodies and have often not been controlled for in earlier CD40 antibody studies. Induction of ADA has frequently been reported with a variety of other human antibodies administered to macaques [69–71]. ADA may result in reduced potency of the CD40 antibodies during subsequent administrations and affect the accuracy of conclusions drawn from sequential dosing, even when using humanized CD40/FcγR mice as the animal model. The ADA we observed after each immunization may have interfered with the efficiency of the boost immunizations. Nevertheless, boosting of antigen-specific T cell responses could be detected after each immunization and especially when a higher antigen dose including Env protein was given. Additional studies using more animals and an optimal dose of antigen, perhaps as well as using a rhesus version of MAB273, are needed to assess the enhancement of T cell responses by the adjuvant effect of MAB273. By comparing the current study with our previous study in which the adjuvant effect of 341G2 was assessed, the range of antigen-specific T cell responses in blood and BAL were comparable [26], although higher dose of the same peptides and higher dose of anti-CD40 antibody were administered i.v. in the previous study. However, co-administration of 341G2 with a TLR-ligand significantly enhanced the T cell responses especially in BAL [26], which exceed the levels detected in the current study. Other agonistic CD40 antibodies have also shown a synergistic adjuvant effect when co-delivered with a TLR-ligand [27, 72, 73]. The addition of a TLR-ligand will be an important next step in future testing of the utility of MAB273.

In summary, our study shows the safety, cell targeting and immunostimulatory properties of this novel agonistic anti-CD40 antibody of IgG1 isotype that is CD40L binding site specific and works independently of FcγR crosslinking. These are distinct features from previously reported agonistic anti-CD40 antibodies and may, therefore, offer new avenues for the adjuvant targeting of the CD40:CD40L pathway.

### Supplementary Information

Below is the link to the electronic supplementary material.

Supplementary Figure 1. Baseline expression of CD40. Related to Figure 1-3. (A). Baseline expression of CD40 on different subsets of human immune cells. Mean fluorescence intensity (MFI) of CD40 is shown (n=7). (B). Baseline expression of CD40 on different subsets of rhesus macaque immune cells. MFI of CD40 is shown (n=13). (C). Gating strategy for phenotyping of innate immune cells. (D). SDS-PAGE shows the kilodalton (kDa) of different products from each step during the preparations of Fab and F(ab’)2. (E). Endotoxin detection assay indicates the endotoxin content level of MAB273, its Fab and F(ab’)2 fragments, CP-870,893 and IgG1-LALA control antibody by the readout of OD value. Standard = standard positive controls. Supplementary Figure 2. Safety monitoring and change of cell frequencies. Related to Figure 4. (A). Safety monitoring by clinical chemistry tests. (B). Safety monitoring by complete blood counts. (C). Cell frequencies normalized by CBC over time. (D). Weight and body temperature change over time. Supplementary Figure 3. Validation of Alexa Fluor 680-conjugated MAB273 and gating strategy. Related to Figure 5. Alexa Fluor 680-conjugated MAB273 labeling test (A), CD40 binding ELISA test (B) and activation test (C) on rhesus PBMCs before tracking immunization. (D). Gating strategy for tracking Alexa Fluor 680-conjugated MAB273. Supplementary Figure 4. MAB273 signal in vivo in NHP and immune cell infiltration. Related to Figure 5 and 6. (A). MAB273+ CD45+ cells normalized by counting beads in peripheral tissues and blood. n=3, mean ± SEM. (B). CD45+ immune cells normalized by weight or counting beads at site of injection and draining LNs. (C). Graphs show cell subsets from (B). n=3, mean ± SEM. (D). Pie charts show proportion of different CD45+ immune cells at the injection site, the primary and secondary draining LNs. The values are the average of three animals. (E). Flow plots of one representative animal show MAB273+ B cells and MAB273+ MDCs at site of injection and draining LNs as well as PBMCs. Supplementary Figure 5. Antibody responses after MAB273 administration in NHP. Related to Figure 7. (A). Levels of MAB273 in NHP plasma over time in immunogenicity study. LLOQ=lower limit of quantification, LLOD=lower limit of detection. n=3. (B). Rhesus anti-human MAB273 IgG titers over time. (C). Volcano plots of differentially expressed genes in blood, “D0” is the day before prime immunization, “D79” is 48h after the second boost. (D). Anti-Env peptides IgG titers over time. (E). Anti-Env protein IgG titers over time. Rhesus plasma from animals immunized six times with Env protein were used as positive control. Supplementary Table 1. Fluorescent staining antibodies used in flow cytometry. Supplementary file1 (PDF 1105 KB)

## Data Availability

The datasets generated during and/or analysed during the current study are available from the corresponding author on reasonable request. The manuscript has data included as electronic supplementary material.
