# Supplementary material for: Cell targeting and immunostimulatory properties of a novel Fcγ-receptor-independent agonistic anti-CD40 antibody in rhesus macaques
Source: Cell Mol Life Sci. 2023 Jun 23;80(7):189. doi: 10.1007/s00018-023-04828-2 (PMC10289945; doi:10.1007/s00018-023-04828-2)
Supplement: Supplementary file 1 — Supplementary Figure 1. Baseline expression of CD40. Related to Figure 1-3. (A). Baseline expression of CD40 on different subsets of human immune cells. Mean fluorescence intensity (MFI) of CD40 is shown (n=7). (B). Baseline expression of CD40 on different subsets of rhesus macaque immune cells. MFI of CD40 is shown (n=13). (C). Gating strategy for phenotyping of innate immune cells. (D). SDS-PAGE shows the kilodalton (kDa) of different products from each step during the preparations of Fab and F(ab’)2. (E). Endotoxin detection assay indicates the endotoxin content level of MAB273, its Fab and F(ab’)2 fragments, CP-870,893 and IgG1-LALA control antibody by the readout of OD value. Standard = standard positive controls. Supplementary Figure 2. Safety monitoring and change of cell frequencies. Related to Figure 4. (A). Safety monitoring by clinical chemistry tests. (B). Safety monitoring by complete blood counts. (C). Cell frequencies normalized by CBC over time. (D). Weight and body temperature change over time. Supplementary Figure 3. Validation of Alexa Fluor 680-conjugated MAB273 and gating strategy. Related to Figure 5. Alexa Fluor 680-conjugated MAB273 labeling test (A), CD40 binding ELISA test (B) and activation test (C) on rhesus PBMCs before tracking immunization. (D). Gating strategy for tracking Alexa Fluor 680-conjugated MAB273. Supplementary Figure 4. MAB273 signal in vivo in NHP and immune cell infiltration. Related to Figure 5 and 6. (A). MAB273+ CD45+ cells normalized by counting beads in peripheral tissues and blood. n=3, mean ± SEM. (B). CD45+ immune cells normalized by weight or counting beads at site of injection and draining LNs. (C). Graphs show cell subsets from (B). n=3, mean ± SEM. (D). Pie charts show proportion of different CD45+ immune cells at the injection site, the primary and secondary draining LNs. The values are the average of three animals. (E). Flow plots of one representative animal show MAB273+ B cells and MAB273+ MDCs at site of [file 18_2023_4828_MOESM1_ESM.pdf]

Supplementary Figure 1

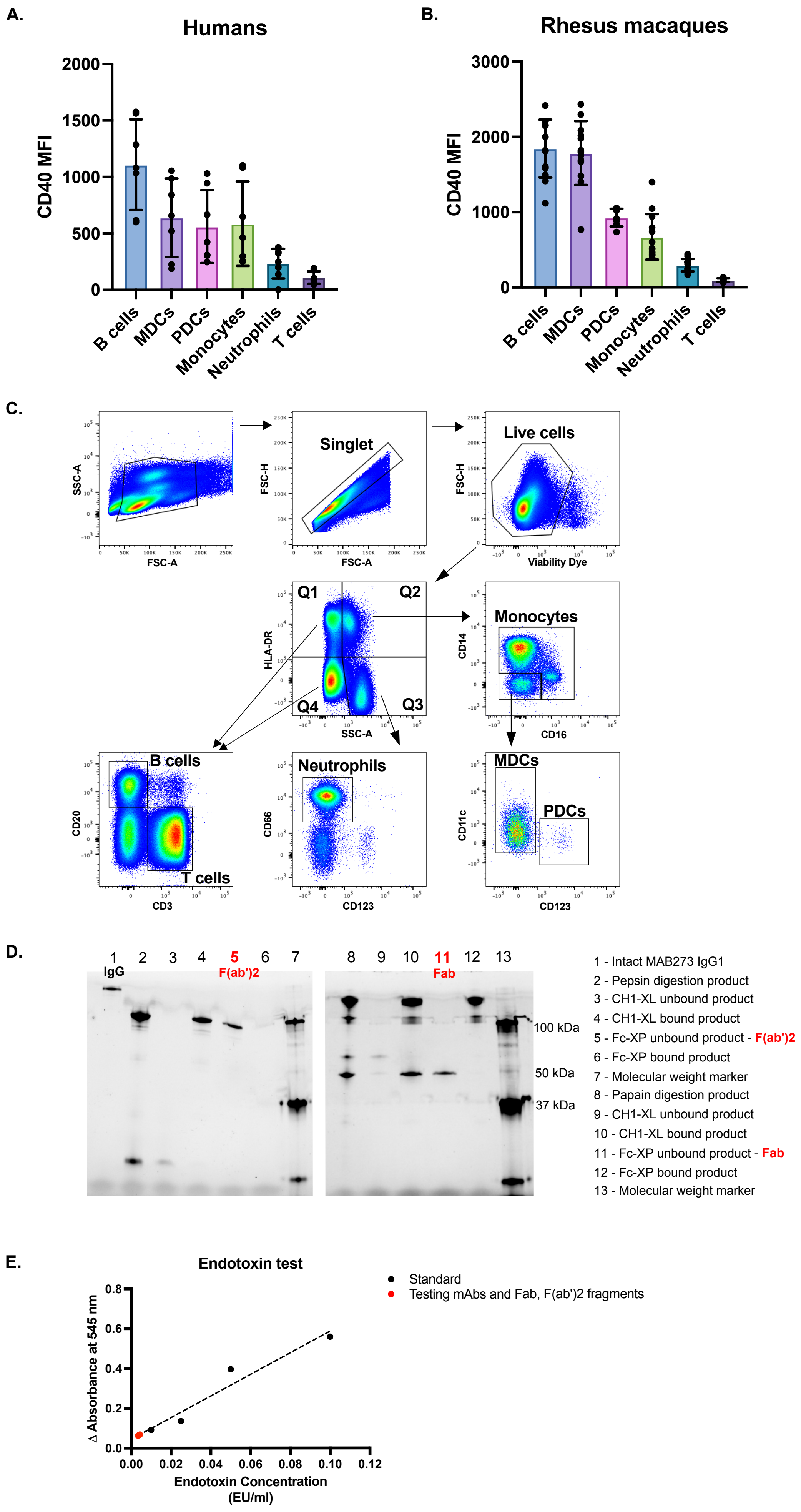

Supplementary Figure 2

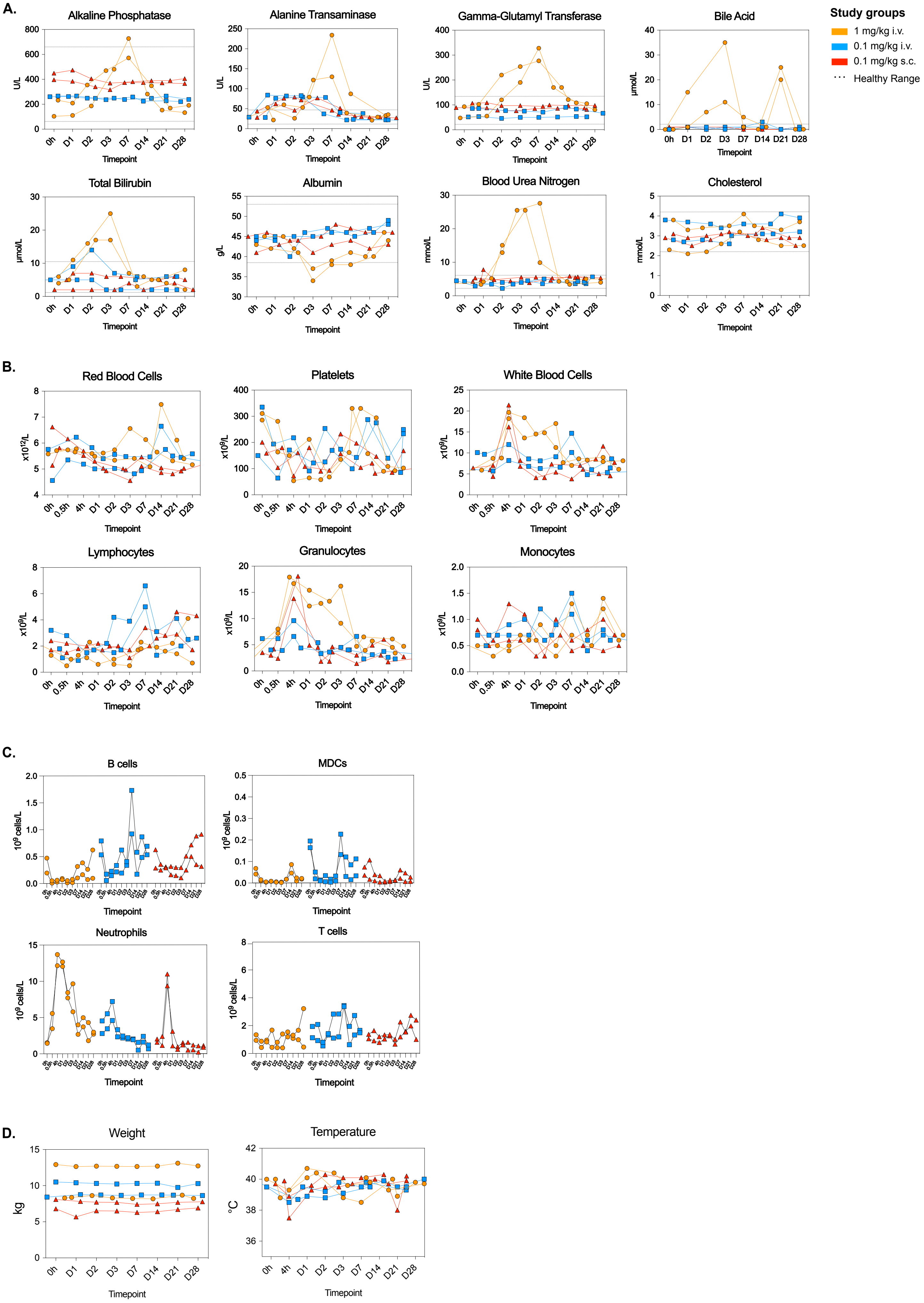

Supplementary Figure 3

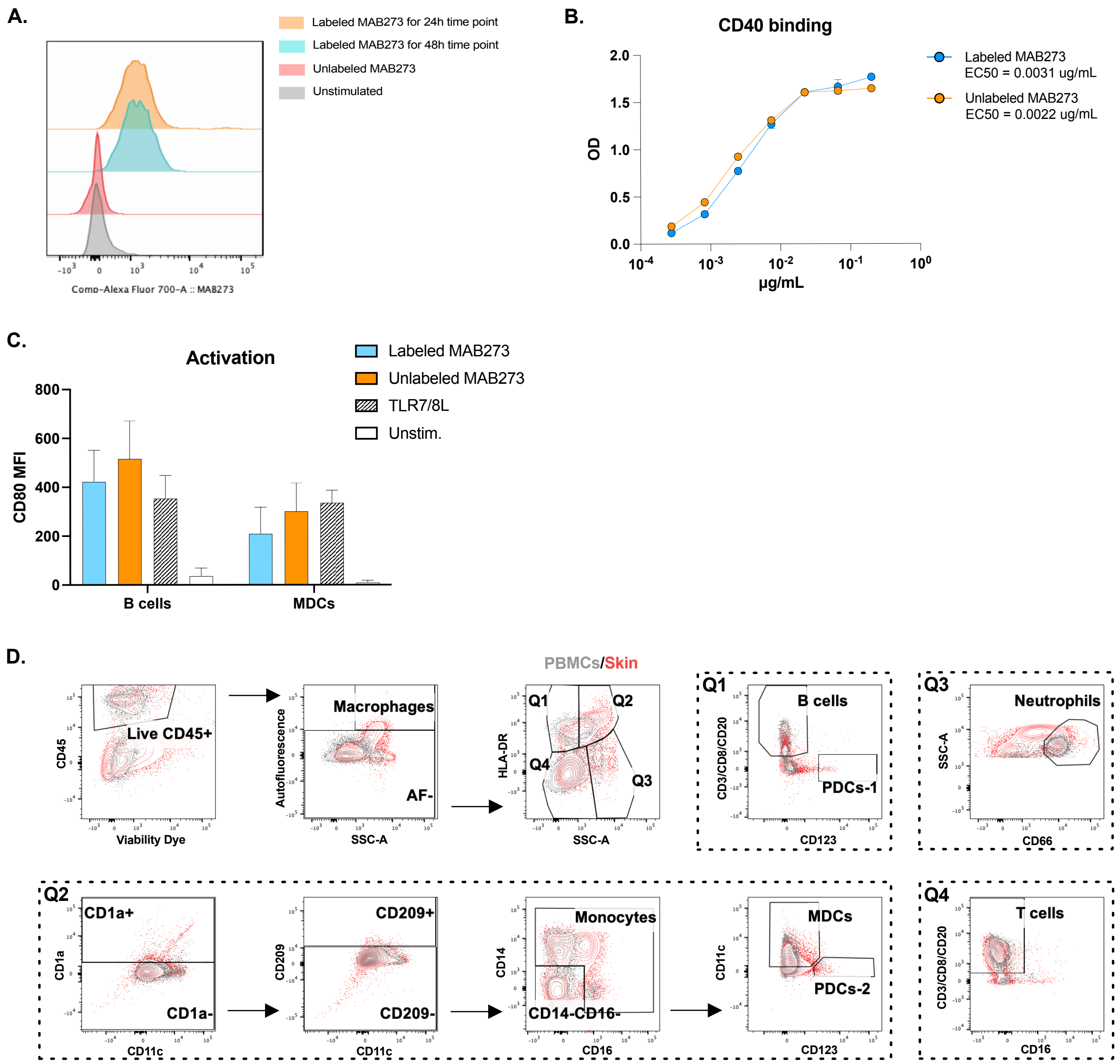

Supplementary Figure 4

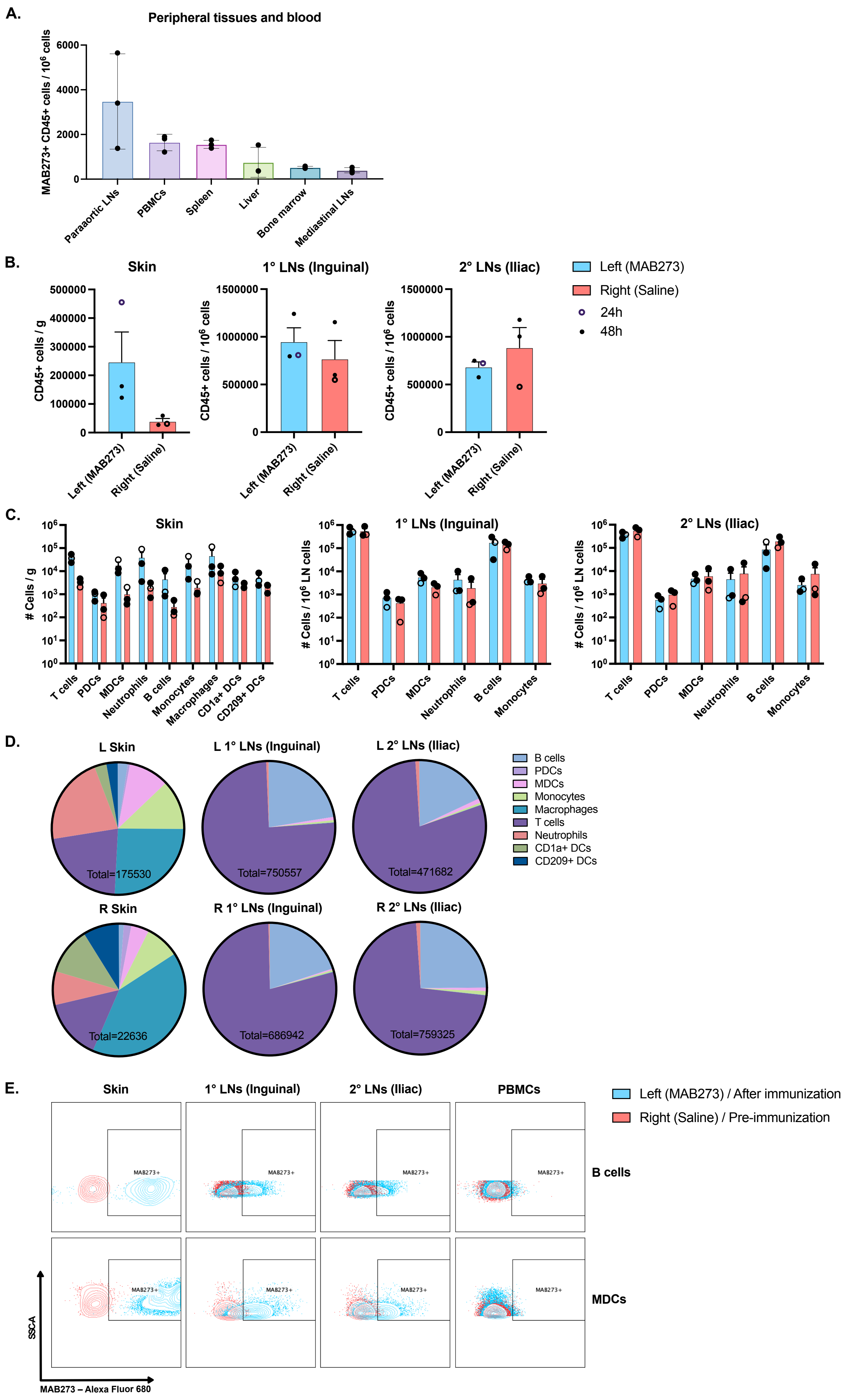

Supplementary Figure 5

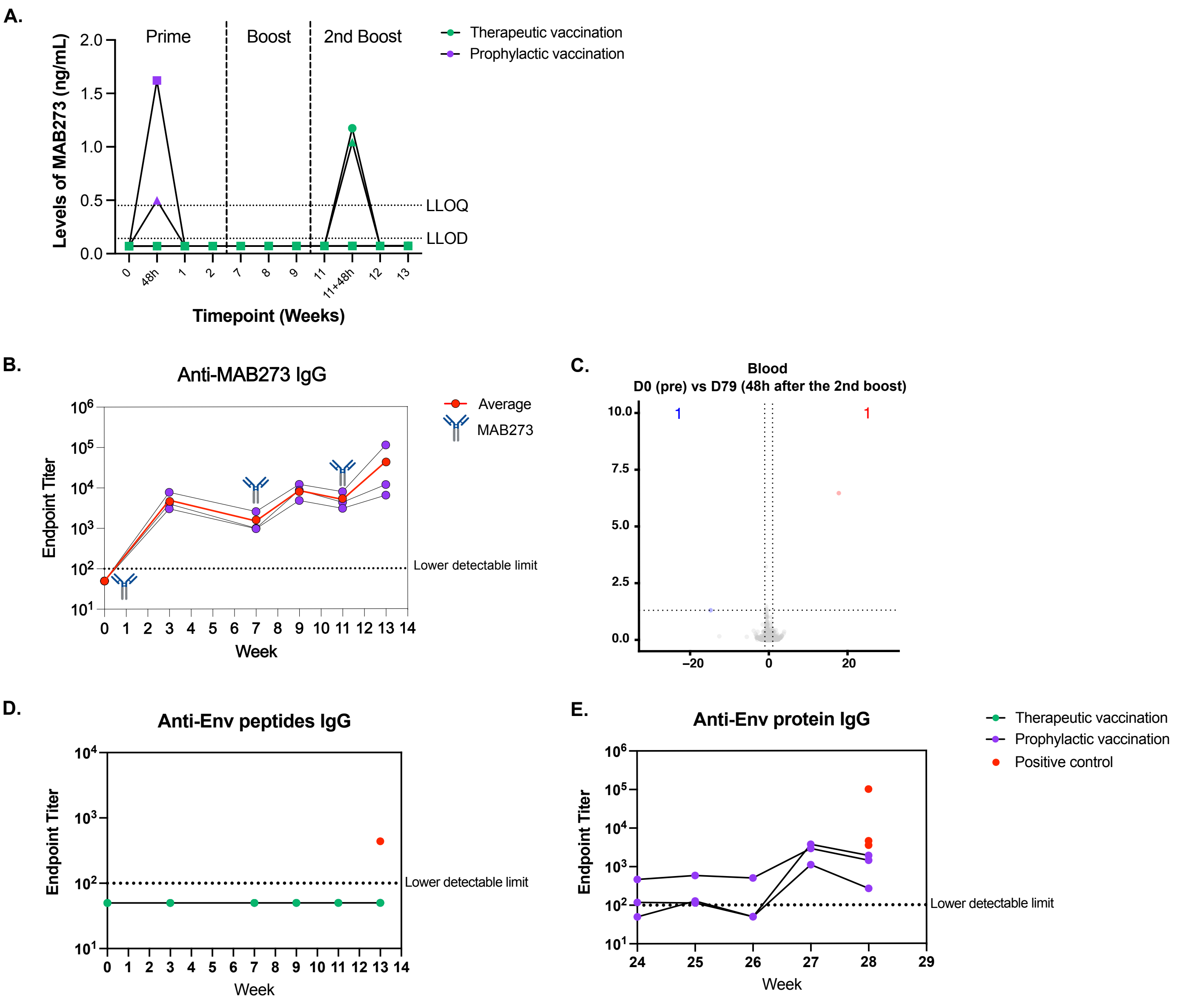

Supplementary Table 1

Antibody	Fluorochrome	Clone	Company
Phenotype of innate immune cells			
CD40	FITC	5C3	Biolegend
NKg2a	PE	Z199	Beckman
CD80	BV421	L307.4	BD
CCR7	PE-Dazzle 594	G043H7	Biolegend
CD123	PerCp-Cy5.5	7G3	BD
CD3	APC-Cy7	SP34-2	BD
CD66	APC	TET2	Miltenyi
CD70	BV786	Ki-24	BD
HLA-DR	BV650	L243	Biolegend
CD11c	PE-Cy7	3.9	Biolegend
CD16	AF700	3G8	BD
CD20	BV605	2H7	Biolegend
CD14	BV510	M5E2	Biolegend
Labeled antibody tracking			
CD1a	PE	SK9	BD
CD209	PerCP-Cy5.5	DCN46	BD
CD40	FITC	5C3	Biolegend
CD11c	PE-Cy7	3.9	Biolegend
CD14	BV711	M5E2	Biolegend
CD66	APC	TET2	Miltenyi
CD45	BV605	D058-1283	BD
CCR7	PE-Dazzle 594	G043H7	Biolegend
CD3	APC-Cy7	SP34-2	BD
CD8	APC-Cy7	RPA-T8	BD
CD20	APC-Cy7	L27	BD
HLA-DR	PE-Cy5.5	Tu36	ThermoFisher
CD123	BV510	6H6	Biolegend
CD80	BV650	L307.4	BD
CD16	BV421	3G8	Biolegend

Antibody	Fluorochrome	Clone	Company
Antigen-specific T cells surface			
OX40	BV510	L106	BD
CCR7	BV786	G043H7	Biolegend
CD103	FITC	2G5	Beckman
CD8a	BV711	RPA-T8	Biolegend
CD4	PE-Cy5.5	S3.5	Invitrogen
CD45RA	BV650	5H9	BD
Antigen-specific T cells intracellular			
4-1BB	APC	4B4-1	BD
IL-2	PE	MQ1-17H12	BD
CD69	ECD	TP1.55.3	Beckman
CD3	APC-Cy7	SP34.2	BD
IFNg	AF700	B27	Biolegend
B cell proliferation			
HLA-DR	PE-Cy5.5	Tu36	ThermoFisher
CD3	APC-Cy7	SP34-2	BD
CD20	BV605	2H7	Biolegend
CD40	FITC	5C3	Biolegend
CD40L competition			
Streptavidin	PE		Biolegend
CD3	APC-Cy7	SP34-2	BD
HLA-DR	PE-Cy5.5	Tu36	ThermoFisher
CD11c	PE-Cy7	3.9	Biolegend
CD20	BV605	2H7	Biolegend
CD14	BV510	M5E2	Biolegend
CD16	BV421	3G8	Biolegend
